# IL-33 as a Novel Serum Prognostic Marker of Intracerebral Hemorrhage

**DOI:** 10.1155/2021/5597790

**Published:** 2021-03-26

**Authors:** Ye Miao, Zhen-xing Zhang, Xu Feng, Wei-ming Sun

**Affiliations:** ^1^Department of Neurosurgery, The First Affiliated Hospital of Jinzhou Medical University, Jinzhou, 121000 Liaoning Province, China; ^2^Department of Breast Surgery, The First Affiliated Hospital of Jinzhou Medical University, Jinzhou, 121000 Liaoning Province, China

## Abstract

**Objective:**

Interleukin 33 (IL-33) is a key cytokine involved in inflammation and oxidative stress. The significance of serum IL-33 levels on the prognosis of patients with intracerebral hemorrhage (ICH) has not been well studied. The purpose of this study is to determine whether there is a relationship between the serum IL-33 level and the prognosis of patients with ICH upon admission.

**Methods:**

A total of 402 patients with confirmed ICH were included in this study. Their demographic data, medical history, laboratory data, imaging data, and clinical scores on admission were collected. At the same time, enzyme-linked immunoassay (ELISA) was used to detect the serum IL-33 levels of patients. The prognosis of patients was evaluated by mRS scale after 3 months, and mRS > 2 was defined as poor prognosis.

**Results:**

Among 402 patients with ICH, the number of patients with good prognosis and poor prognosis after 3 months was 148 and 254, respectively. Compared with the ICH group with poor prognosis, the ICH group with good prognosis had lower baseline NHISS scores (*p* = 0.039) and hematoma volume (*p* = 0.025) and higher GCS scores (*p* < 0.001) and serum IL-33 levels (*p* < 0.001). The results of linear correlation analysis showed that serum IL-33 levels were significantly negatively correlated with baseline NHISS scores (*r* = −0.224, *p* = 0.033) and hematoma volume (*r* = −0.253, *p* = 0.046) but were significantly positively correlated with baseline GCS scores (*r* = 0.296, *p* = 0.020). The receiver operating characteristic curve (ROC) analysis showed that the sensitivity and specificity of serum IL-33 level in evaluating the prognosis of ICH were 72.1% and 74.3%, respectively. A cut-off value of serum IL-33 level < 109.3 pg/mL may indicate a poor prognosis for ICH.

**Conclusions:**

Serum IL-33 level on admission may be a prognostic indicator of ICH, and its underlying mechanism needs further study.

## 1. Introduction

Intracranial hemorrhage (ICH) usually refers to a neurological syndrome in which blood enters the brain tissue secondary to hypertension or other vascular diseases [1]. ICH is the second most common type of stroke, accounting for about 10%-20% of all strokes [2–4]. In developed countries such as Europe and the United States, due to the effective control of hypertension, the incidence of ICH has been on a downward trend in recent years, while in developing countries, its incidence is still high [5]. A study pointed out that the annual incidence of ICH is 4.2 per 100,000 in Caucasians, 22.9 per 100,000 in blacks, 19.6 per 100,000 in Hispanics, and 51.8 per 100,000 in Asians [6]. According to Global Burden of Disease reports, there were 62.8 million disability-adjusted life years (DALY) lost due to ICH in 2010, of which 86% were in low- and middle-income countries [7]. Although our research on ICH has made great progress in the past few decades, its disability rate and fatality rate are still relatively high. So far, there is still no cure for stroke, which has brought a heavy burden to society [8–10]. Looking for prognostic biomarkers and potential therapeutic targets of ICH is of great significance to the prevention and treatment of ICH.

Interleukin-33 (IL-33) is an important cytokine that regulates innate immune response [11]. In 2005, it was discovered that it can specifically bind to IL-1 receptor ST2, so IL-33 is recognized as one of the IL-1 family members [12]. IL-33 is composed of 270 and 266 amino acids in humans and mice and has a molecular weight of 30-34 kDa [13]. It appears in various tissues and organs of the body and is mainly expressed in endothelial cells, epithelial cells, and fibroblast-like cells [14]. The wide distribution of IL-33 and the complexity of its structure make it possible to participate in multisystem diseases.

IL-33 is identified as a dual cytokine by exerting its protective and harmful effects as a support or anti-inflammatory effect. Thus, it seems to be a double-edged sword [15]. IL-33 has been widely reported in Alzheimer's disease (AD), multiple sclerosis (MS), and intracranial infection [16–18], but its relationship with ICH is relatively rare. The purpose of our current study was to determine the relationship between serum IL-33 levels and the prognosis of ICH, which were aimed at providing a theoretical basis for the treatment of ICH.

## 2. Methods

### 2.1. Study Population

**Figure 1:**
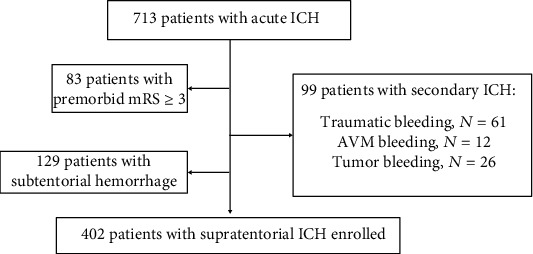
Flow chart for patients' selection. A total of 402 ICH patients were included in the study. ICH: intracranial hemorrhage; mRS: the Modified Rankin Scale; AVM: arteriovenous malformations.

A total of 713 ICH patients who attended the First Affiliated Hospital of Jinzhou Medical University from January 1, 2018, to December 31, 2020, were included in the study. The inclusion criteria of ICH are (1) hospitalized patients within 24 hours of onset and (2) computed tomography (CT) diagnosed as ICH. Exclusion criteria for ICH are (1) mRS ≥ 3 before admission; (2) nonspontaneous ICH caused by trauma, tumor, and arteriovenous malformation (AVM); (3) ICH patients who needed surgical intervention; (4) subtentorial hemorrhage; and (5) severe liver and kidney dysfunction. The flow chart for patients' selection is shown in Figure 1. The study was approved by the local ethics committee, and all patients and their families allowed us to use these data for scientific research.

### 2.2. Baseline Characteristic Collection

The age and gender information of all subjects was collected upon admission. At the same time, the patient's past chronic medical history, such as hypertension, diabetes, hyperlipidemia, and coronary heart disease, was collected. A sphygmomanometer was used to measure the patient's systolic and diastolic blood pressure at rest. Standardized laboratory methods are used to detect the biochemical indicators of patients: TG, LDL-C, HbA1c, FBG, APTT, and INR.

### 2.3. Neurological Assessment

The Glasgow Coma Scale (GCS) was firstly described in 1974 by Teasdale et al. [19], which was widely used to assess the patient's level of consciousness on admission. GCS evaluates a person's level of consciousness based on three aspects: eye, verbal, and motor. The GCS score is between 3 and 15 points, which is inversely related to the level of consciousness.

The National Institutes of Health Stroke Scale (NIHSS) is used to determine the degree of neurological impairment on admission. NIHSS consists of 11 projects, each of which scores a specific ability between 0 and 4 points. The total score of NIHSS is between 0 and 42 points. The higher the score, the more severe the damage [20].

### 2.4. Imaging Analysis

Upon admission, all patients underwent head CT to confirm the diagnosis of ICH. At the same time, commercial Analyze Direct 11.0 software (Analyze Direct, Overland Park, MS, USA) was used to determine the bleeding volume of ICH. All head CT images are analyzed by experienced imaging doctors who are blinded to the detailed research protocol.

### 2.5. Serum IL-33 Level Determination

The peripheral venous blood of all ICH patients in the fasting state was collected by the nurse within 24 hours after admission. All venous blood is allowed to stand at room temperature for 20 minutes, and the serum is collected after high-speed centrifugation. The serum IL-33 level was measured using commercial ELISA reagents (Abcam, Cambridge, MA, USA). The operation of ELISA is strictly in accordance with the product specification and previous reports [21–23].

### 2.6. Follow-Up

After 3 months of ICH, all patients were routinely followed up, and the prognosis was assessed using the Modified Rankin Scale (mRS) [24]. The mRS is a commonly used scale to measure the degree of disability or dependence on daily activities in patients with stroke or other neurological disorders. The scale was originally proposed in 1957 by Dr. John Rankin of Stobhill Hospital in Glasgow, Scotland. It has become the most widely used prognostic assessment scale in stroke clinical trials. The score of mRS ranges from 0 to 6 points. In this study, we defined mRS ≥ 3 as poor prognoses at 3 months after ICH and mRS < 3 as good prognoses.

### 2.7. Statistical Analysis

All statistics are completed using SPSS 20.0 (SPSS Inc., Chicago, IL, USA). Continuous variables are expressed as mean ± standard deviation, and categorical variables are expressed as frequency (%). The comparison between two categorical variables uses the chi-square test, and the comparison between two continuous variables uses the *t*-test. In order to clarify the relationship between serum IL-33 levels and baseline characteristics in ICH patients, we adopted Pearson correlation analysis. The receiver operating characteristic curve (ROC) was used to analyze and calculate the cutoff point of serum IL-33 level predicting the poor prognosis of ICH patients. If the *p* value is less than 0.05, it is considered statistically significant.

## 3. Results

### 3.1. Baseline Characteristics

After strict screening, a total of 402 ICH patients entered the final study. According to the follow-up results of the mRS score after 3 months, we divided them into a good prognosis group (mRS < 3) and a poor prognosis group (mRS ≥ 3). The baseline characteristics according to 3-month prognosis in patients with ICH are shown in Table 1.

We compared the demographics (age and gender), chronic diseases (hypertension, DM, hypertension, and CHD), and clinical indicators (SBP, DBP, TG, LDL-C, HbA1c, FBG, APTT, and INR) of the two groups. The results showed that there was no significant statistical difference between the above indicators of the two groups (*p* > 0.05).

The degree of neurological deficit at the onset of ICH patients was assessed by NHISS and GSC scales. The NHISS scores of the good prognosis group and the poor prognosis group were 11.3 ± 3.6 and 12.1 ± 3.8, respectively. There was a significant statistical difference between the two groups (*p* = 0.039). The GSC scores of the good prognosis group and the poor prognosis group were 14.1 ± 0.7 and 13.7 ± 0.6, respectively. There is a significant statistical difference between the two groups (*p* < 0.001).

We also compared the bleeding volume of the two groups of patients with ICH. The hematoma volume of the good prognosis group and the poor prognosis group was 12.5 ± 4.9 mL and 13.7 ± 5.3 mL, respectively. There was a significant statistical difference between the two groups (*p* = 0.025).

### 3.2. Serum IL-33 Level Determination

**Figure 2:**
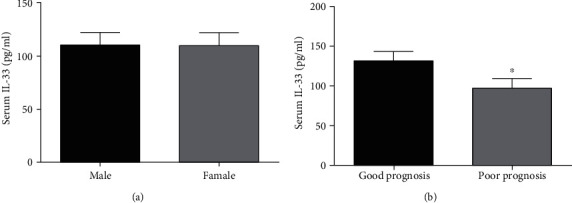
Serum IL-33 levels according to gender and prognosis in patients with ICH: (a) serum IL-33 levels according to gender; (b) serum IL-33 levels according to prognosis. IL-33: interleukin 33; ICH: intracranial hemorrhage. Compared to good prognosis group, ^∗^*p* < 0.05.

The results of ELISA showed that the serum IL-33 levels of the good prognosis group and the poor prognosis group of ICH patients were 132.3 ± 12.6 pg/mL and 97.5 ± 11.4 pg/mL, respectively. There is a significant difference between the two groups (*p* < 0.001). We also compared the serum IL-33 levels in patients with ICH according to genders, and the results showed that gender is not a significant factor affecting serum IL-33 levels (*p* > 0.05). The above results are shown in Figure 2.

**Figure 3:**
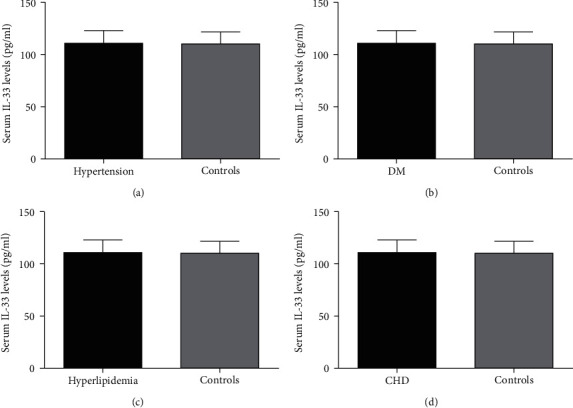
Serum IL-33 levels in different subgroups of ICH patients: (a) serum IL-33 levels in patients with or without hypertension; (b) serum IL-33 levels in patients with or without DM; (c) serum IL-33 levels in patients with or without hyperlipidemia; (d) serum IL-33 levels in patients with or without CHD. IL-33: interleukin 33; ICH: intracranial hemorrhage; DM: diabetes mellitus; CHD: coronary heart disease.

We further compared the differences in serum IL-33 levels in different subgroups of ICH patients. The results showed that there was no significant difference in serum IL-33 levels between the hypertensive group and the nonhypertensive group (*p* > 0.05). Similar results appeared in ICH patients with or without DM, hyperlipidemia, and CHD (*p* > 0.05). The above results suggest that chronic diseases such as hypertension, DM, hyperlipidemia, and CHD are not interfering factors that affect the serum IL-33 levels in ICH patients. The results of serum IL-33 levels in different ICH subgroups are shown in Figure 3.

### 3.3. Linear Correlation Analysis

The results of correlation analysis showed that serum IL-33 levels in ICH patients were not significantly correlated with age, gender, SBP, DBP, TG, LDL-C, HbA1c, FBG, APTT, and INR (*p* > 0.05). However, serum IL-33 levels in ICH patients are significantly negatively correlated with NHISS scores (*r* = −0.224, *p* = 0.033) and hematoma volume (*r* = −0.253, *p* = 0.046) and positively correlated with GCS scores (*r* = 0.296, *p* = 0.020). The results of linear correlation analysis are shown in Table 2.

### 3.4. ROC Analysis

**Figure 4:**
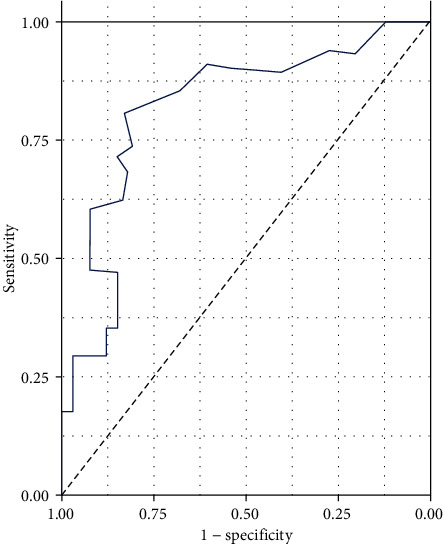
ROC analysis of IL-33 levels for the prognosis of ICH. The sensitivity and specificity of serum IL-33 in predicting the prognosis of ICH were 72.1% and 74.3%, respectively. ROC: receiver operating curve; IL-33: interleukin 33; ICH: intracranial hemorrhage.

In order to clarify the diagnostic value of serum IL-33 levels for the prognosis of ICH, we performed ROC analysis, as shown in Figure 4. The sensitivity and specificity of serum IL-33 in diagnosing poor prognosis of ICH were 72.1% and 74.3%, respectively. The cutoff value of serum IL-33 for diagnosing poor prognosis of ICH is 109.3 pg/mL.

## 4. Discussions

Our current study explored the difference in serum IL-33 levels between ICH patients with good prognosis and poor prognosis. The results showed that serum IL-33 levels in ICH patients with good prognosis were significantly higher than those with poor prognosis. The results of linear analysis further showed that the level of serum IL-33 is related to NHISS score, GCS score, and hematoma volume, and the latter is a traditional indicator for evaluating the prognosis of ICH. In order to determine the diagnostic value of serum IL-33 levels for the prognosis of ICH, we performed ROC analysis and found that serum IL-33 may be a potential predictor of the prognosis of ICH, with high sensitivity and specificity.

IL-33 is a member of the IL-1 family, which can exhibit proinflammatory and anti-inflammatory properties according to the type of disease itself. In the body [25], IL-33 is mainly expressed in endothelial cells and epithelial cells, and its function is regulated by the interaction with multiple receptors such as ST2 [26]. In addition to inflammation, IL-33 is also considered to be closely related to oxidative stress in recent years. The study of Uchida et al. showed that the balance between oxidative stress and antioxidant response plays a key role in controlling the release of IL-33 in airway epithelial cells [27]. The study by Aizawa et al. reached a similar conclusion. Oxidative stress participates in the expression of IL-33 in airway epithelial cells through the MAPK signaling pathway, suggesting that oxidative stress may be a potential target for the treatment of COPD [28]. Interestingly, serum IL-33 levels in patients with chronic heart failure are elevated, and it has been proven to have antioxidant effects. IL-33 plays an important role in inflammation and oxidative stress, making it possible to participate in a variety of pathophysiological processes [29].

In recent years, the role of IL-33 in neurological diseases has attracted intense attention. IL-33 can reverse the synaptic plasticity damage and memory deficits in APP/PS1 mice, and its mechanism may be to promote the differentiation of microglia into an anti-inflammatory phenotype and reduce soluble A*β* levels and amyloid plaque deposition [30]. Not only in animal experiments, Yu and his colleagues found that IL-33 gene mutations affect the susceptibility of Han people to late-onset AD, further confirming that IL-33 is involved in the pathogenesis of AD [31]. A study in the United States found that the levels of IL-33 in the periphery and the center of patients with multiple sclerosis (MS) increased, suggesting that IL-33 is an important factor involved in MS [32]. In addition, IL-33 can attenuate brain parenchymal damage caused by encephalitis by regulating iNOS [32]. Interestingly, IL-33 is also believed to be involved in the onset of schizophrenia, traumatic encephalopathy, and glioma [33–35].

The involvement of IL-33 in the course of ischemic stroke has been widely reported. The study of Li et al. pointed out that there is a correlation between the common variants of IL-33 and the reduced risk of ischemic stroke in the Chinese Han population [36]. Similarly, Li and his colleagues found that IL-33 improves ischemic brain injury by promoting Th2 response and inhibiting Th17 response [36]. Interestingly, earlier studies pointed out that IL-33 could attenuate the occurrence and development of atherosclerosis by inducing IL-5 and ox-LDL antibodies [37]. In addition, a recent Sino-US joint study found that serum IL-33 was a new biomarker for the diagnosis and prognosis of acute ischemic stroke [38].

In addition to ischemic stroke, the relationship between IL-33 and ICH has gradually been reported. A clinical study from Chongqing found that IL-33 is a new biomarker that predicts hemorrhagic transformation of acute ischemic stroke [39]. In addition, animal studies have shown that IL-33 has a neuroprotective effect on cerebral hemorrhage, and its mechanism may be related to the selective activation of M2 type microglia, reducing inflammation, apoptosis, and autophagy [40, 41]. Our current research is consistent with the above results, suggesting that high levels of IL-33 may reduce brain damage after ICH. However, another study found that increased IL-33 concentration was associated with poor prognosis of aneurysmal subarachnoid hemorrhage (SAH) [25]. These results suggest that first, SAH is a special type of cerebral hemorrhage, and its pathogenesis is special; second, IL-33 may play a dual role of anti-inflammatory or anti-inflammatory depending on the diseases.

Except for serum biomarkers, many easy-to-use biomarkers also have been reported to predict clinical prognosis in recent years, such as noncontrast CT biomarkers. A noncontrast CT scan is a computer tomography scan that does not use a special dye during the scan to make the organs more visible [42]. This technology provides a fast, noninvasive method that does not require the use of contrast agents to diagnose the disease, so it has received widespread attention in recent years. In order to explore the ability of quantitative radiomics extracted from noncontrast-enhanced CT images to distinguish arteriovenous malformation- (AVM-) related cerebral hemorrhage from other causes of cerebral hemorrhage, Zhang et al. did related research. Their research found that the machine learning model of radiomic features extracted from noncontrast CT images can accurately distinguish AVM-related intracranial hematomas and hematomas caused by other causes [43]. Researchers from the same unit further conducted a clinical study of noncontrast CT markers to predict intracranial hematoma. They found that noncontrast CT helps to identify high-risk active ICH in time, with high specificity and accuracy, but limited sensitivity [44]. In addition, Li and his colleagues found that blend sign and black hole sign can be easily identified on nonenhanced CT, which has a high degree of specificity in predicting the growth of hematoma [45, 46]. The team also found that the island sign is a reliable CT imaging marker that can independently predict hematoma expansion and poor prognosis in patients with cerebral hemorrhage, and it may be a potential marker for therapeutic intervention [47]. Interestingly, in recent years, researchers have proposed that gap analysis can be used as an excellent prognostic model [48], and its combination with the biomarker IL-33 may provide a new methodological reference for improving the prognosis of ICH.

Our research has some limitations. Firstly, we are a single-center study with a small sample, which is obviously regional; secondly, we have monitored the serum IL-33 level at the time of admission but did not do its dynamic monitoring; thirdly, we have not collected relevant information about smoking and drinking in ICH patients. These lifestyle habits may affect the level of serum IL-33; fourthly, we could not able to assess IL-33 in different ICH etiologies due to sample size. Finally, we did not monitor the long-term prognosis of ICH and only did a short-term follow-up 3 months after the onset. However, our research also has advantages. So far, there is no report about the relationship between serum IL-33 and the prognosis of ICH.

## 5. Conclusions

Low serum IL-33 levels may indicate a poor prognosis for ICH. A large sample and multicenter study is needed to further confirm this predictive effect of IL-33 and provide a potential target for the prevention and treatment of ICH.

## Figures and Tables

**Table 1:** Baseline characteristics according to 3-month prognosis in patients with ICH.

Characteristics on admission	3-month prognosis		*p* values
	Good (*n* = 148)	Poor (*n* = 254)	
Age (years)	64.2 ± 5.6	64.8 ± 5.3	0.284
Gender, male/female	91/57	165/89	0.485
Hypertension (*n*, %)	118	200	0.814
DM (*n*, %)	37	49	0.178
Hyperlipidemia (*n*, %)	75	122	0.609
CHD (*n*, %)	14	29	0.540
SBP (mmHg)	176.3 ± 12.5	177.6 ± 11.2	0.283
DBP (mmHg)	102.9 ± 10.3	103.2 ± 9.7	0.770
TG (mmol/L)	5.0 ± 0.8	5.1 ± 1.0	0.300
LDL-C (mmol/L)	2.8 ± 0.4	2.9 ± 0.6	0.072
HbA1c (mmol/L)	6.4 ± 0.7	6.3 ± 0.6	0.131
FBG (mmol/L)	6.8 ± 1.1	6.9 ± 1.2	0.407
APTT	28.6 ± 4.2	28.8 ± 4.3	0.650
INR	1.67 ± 0.24	1.69 ± 0.27	0.456
NHISS scores	11.3 ± 3.6	12.1 ± 3.8	0.039
GCS scores	14.1 ± 0.7	13.7 ± 0.6	<0.001
Hematoma volume (mL)	12.5 ± 4.9	13.7 ± 5.3	0.025
Serum IL-33 (pg/mL)	132.3 ± 12.6	97.5 ± 11.4	<0.001

ICH: intracranial hemorrhage; DM: diabetes mellitus; CHD: coronary heart disease; SBP: systolic pressure; DBP: systolic pressure; TG: triglyceride; LDL-C: low-density lipoprotein cholesterol; HbA1c: glycated hemoglobin; FBG: fasting blood glucose; APTT: activated partial thromboplastin time; INR: international normalized ratio; NHISS: National Institutes of Health Stroke Scale; GCS: Glasgow Coma Scale; IL-33: interleukin 33.

**Table 2:** The association between serum IL-33 levels and clinical characteristics.

Clinical characteristics	*r*	*p*
Age	-0.211	0.419
SBP (mmHg)	0.139	0.263
DBP (mmHg)	0.247	0.615
TG (mmol/L)	0.401	0.317
LDL-C (mmol/L)	0.193	0.108
HbA1c (mmol/L)	0.308	0.225
FBG (mmol/L)	0.272	0.531
APTT	0.285	0.378
INR	0.360	0.314
NHISS scores	-0.224	0.033
GCS scores	0.296	0.020
Hematoma volume (mL)	-0.253	0.046

IL-33: interleukin 33; SBP: systolic pressure; DBP: systolic pressure; TG: triglyceride; LDL-C: low-density lipoprotein cholesterol; HbA1c: glycated hemoglobin; FBG: fasting blood glucose; APTT: activated partial thromboplastin time; INR: international normalized ratio; NHISS: National Institutes of Health Stroke Scale; GCS: Glasgow Coma Scale.

## Data Availability

The data used to support the findings of this study are available from the corresponding author upon request.
